# Profiles of biliary microbiota in biliary obstruction patients with *Clonorchis sinensis* infection

**DOI:** 10.3389/fcimb.2023.1281745

**Published:** 2023-12-18

**Authors:** Rui Chen, Xiang Li, Jian Ding, Jie Wan, Xueli Zhang, Xu Jiang, Shanshan Duan, Xinyi Hu, Yannan Gao, Beibei Sun, Xi Lu, Ruifeng Wang, Yang Cheng, Xiaoli Zhang, Su Han

**Affiliations:** ^1^ Jiangnan University Medical Center, Jiangnan University, Wuxi, China; ^2^ Central Laboratory, First Affiliated Hospital, School of Medicine, Zhejiang University, Hangzhou, China; ^3^ Department of Parasitology, Harbin Medical University, Harbin, China; ^4^ Department of Public Health and Preventive Medicine, Wuxi School of Medicine, Jiangnan University, Wuxi, China; ^5^ Department of Gastroenterology, The First Affiliated Hospital of Harbin Medical University, Harbin, China; ^6^ Department of Gastroenterology, The Fourth Affiliated Hospital of Harbin Medical University, Harbin, China

**Keywords:** biliary obstruction, *Clonorchis sinensis*, biliary microbiota, sequencing, bile

## Abstract

**Background:**

*Clonorchis sinensis* (*C. sinensis)* is a epidemiologically significant food-borne parasite, causing several hepatobiliary diseases. Biliary microbiota community structure might be influenced by infection with pathogens. However, the biliary microbiome of biliary obstruction patients infected with *C. sinensis* is still an unexplored aspect.

**Methods:**

A total of 50 biliary obstruction patients were enrolled, including 24 infected with *C. sinensis* and 26 non-infected subjects. The bile samples were collected by Endoscopic Retrograde Cholangiopancretography. Biliary microbiota alteration was analyzed through high-throughput 16S ribosomal RNA (rRNA) gene sequencing.

**Results:**

Our findings revealed that there was significant increase in both richness and diversity, as well as changes in the taxonomic composition of the biliary microbiota of *C. sinensis* infected patients. At the phylum level, *C. sinensis* infection induced Proteobacteria increased and Firmicutes reduced. At the genus level, the relative abundance of *Pseudomonas* and *Staphylococcus* increased significantly, while *Enterococcus* decreased prominently in infected groups (*P* < 0.05). The PICRUSt analysis further showed remarkably different metabolic pathways between the two groups.

**Conclusion:**

*C. sinensis* infection could modify the biliary microbiota, increasing the abundance and changing the phylogenetic composition of bacterial in biliary obstruction patients. This study may help deepen the understanding of the host-biliary microbiota interplay with *C. sinensis* infection on the background of biliary obstruction and provide new insights into understanding the pathogenesis of clonorchiasis.

## Background

*Clonorchis sinensis* is an important foodborne parasite. Human infected through eating raw or undercooked fish contain *C. sinensis* metacercariae. *C. sinensis* adults parasitize in the intrahepatic bile duct and lead to clonorchiasis. Clonorchiasis seriously afflicts more than 35 million people globally and has become a serious public health in endemic regions (Lun et al., 2005). Although acute infection is usually asymptomatic, chronic clonorchiasis is related to many hepatobiliary diseases, such as cholecystitis, cholangitis, periductal fibrosis and even cholangiocarcinoma (CCA) (Tang et al., 2016; Brindley et al., 2021). Especially, the size of *C. sinensis* is similar to human bile duct, biliary obstruction with *C. sinensis* infection can cause bile stasis and biliary pressure increased, eventually promoting the development of CCA (Prueksapanich et al., 2018). CCA is an aggressive and heterogeneous malignancy of the biliary tree, which is considered arising from a complex interaction between host-specific genetic background and multiple risk factors including parasite infection (Khan et al., 2019). *C. sinensis* has been classified as group I biological carcinogen by the International Agency for Research on Cancer in 2009 (Bouvard et al., 2009). However, influence of *C. sinensis* infection on the biliary microbiome of biliary obstruction remains obscure.

The gut microbiota plays significant impacts on host metabolism, immunology and the behavior (Thaiss et al., 2016; Chen et al., 2017). The highthroughput sequencing method is a powerful tool for analyzing microbial community structure (Liu et al., 2020). To date, the gut microbiota has a potential influence on kinds of hepatobiliary diseases. The gut microbiota could participate in the enterohepatic bile acids recycling process, keeping the balance of complex bacterial communities and biliary system (Wang et al., 2017; Tripathi et al., 2018). Furthermore, microbial products have been identified to be possible triggers for chronic and acute inflammatory biliary illnesses (Negm et al., 2010; Sayin et al., 2013; Verdier et al., 2015; Albillos et al., 2020).

In a normal biliary system, bile has traditionally been considered to be sterile; while some studies confirmed the existence of biliary microbiota in healthy and diseased states (Choi et al., 2021). For instance, studies in pigs revealed that the gallbladder ecosystem contained bacteria including the Firmicutes, Bacteroidetes and Proteobacteria phyla (Jimenez et al., 2014). Based on 16S rRNA gene profiling, the Firmicutes, Bacteroidetes, and Actinobacteria phyla in human intact gallbladder bile has been verified (Molinero et al., 2019). Recently, biliary microbiota dysbiosis has been related to the development of hepatobiliary illnesses such as recurrent gallstones, primary sclerosing cholangitis and extrahepatic cholangiocarcinoma. For instance, compared with non-biliary stones, recurrent gallstones patients were identified with significantly lower bacterial diversity in the biliary system, as well as higher Proteobacteria phylum and lower Bacteroidetes, which indicating a substantial link between recurrent gallstones and biliary microbial dysbiosis (Ye et al., 2020). *Streptococcus* genus was identified to be positively linked with illness severity, playing a pathogenic role in the progression of primary sclerosing cholangitis (Pereira et al., 2017). There is increasing evidence supporting that biliary microbiota has potential influence on human physiology (Chen et al., 2020).

Adult *C. sinensis* inhabit the biliary and can cause the alterations of liver functions and the biliary environment and which, in turn, may modify the composition of the biliary microbiota (Qian et al., 2016). Indeed, the dynamic microbiome has been identified in fecal from human or rat’s models with *C. sinensis* infection. However, it is still unknown about the profiles of biliary microbiota in biliary obstruction patients with *C. sinensis* infection. Therefore, 16S rRNA gene sequencing was performed using bile samples from 24 C*. sinensis* infected and 26 non-infected patients with biliary obstruction, in order to characterize the biliary microbial community. Overall, our study first time explored the relationship between biliary microbiota profile and *C. sinensis* infection on the background of biliary obstruction. These results may be helpful for providing light on the underlying mechanisms of the host-biliary microbiota interplay with *C. sinensis* infection, deepening understanding of the pathogenesis of clonorchiasis.

## Methods

### Ethics statement

This study’s all protocols and procedures followed the ethical criteria specified in the 1975 Declaration of Helsinki, as indicated by a priori. All individuals provided written informed permission before participating in this study. Harbin Medical University’s Ethics Committee approved this study. All experiments were performed in compliance with the established guidelines and regulations.

### Subjects enrollment and sample collection

A total of 45 diagnosed biliary obstruction patients with *C. sinensis* infection and 50 non-infected biliary obstruction patients in the first and fourth affiliated Hospital of Harbin Medical University (Harbin, China) were recruited in this study. The biliary obstruction patients were diagnosed according to the clinical typical symptoms, blood sample tests, B ultrasound, computed tomography (CT) or magnetic resonance cholangiography (MRCP), which were subsequently confirmed by endoscopic retrograde cholangiopancreatography (ERCP). The study inclusion criteria were as follows: (1) diagnosis of bile duct obstruction with evidence by abdominal imaging (B ultrasound, MRCP and ERCP; (2) the need for endoscopic retrograde cholangiopancreatography (ERCP) for bile duct decompression; (3) a naïve ampulla and (4) older than 20 years old.

The exclusion criteria included: (1) clinical data are incomplete, (2) stool inspection of eggs found other parasitic eggs, (3) malignant bile duct obstruction due to acute suppurative cholangitis, pancreatic cancer and cholangiocarcinoma etc., (4) antibiotic use in the prior 6 months, (5) long-term use of probiotics or prebiotics, (6) long-term probiotics or prebiotics use, (7) chronic hepatitis or liver disease with functional damage, (8) active viral, bacterial, or fungal infections, (9) additional illnesses like any kind of malignancy, or uncontrolled chronic conditions involving the heart, liver, kidney and lung. The detailed demographic characteristics and medical history (diabetes, hypertension, coronary heart disease, hypercholesterolemia, constipation, and diarrhea) were retrieved from hospital medical records.

After rigorous screening based on inclusion and exclusion criteria, and removal of low-quality samples, 24 biliary obstruction patients with *C. sinensis* infection and 26 sex, age, dietary pattern and rural life history similar patients with biliary obstruction alone were enrolled for biliary microbiota analysis. All patients were Han Chinese and born in Northeastern, with similar geographic areas and eating habits, without special dietary habits.

The bile samples from all patients were obtained after ERCP. A total of 5-10 ml bile was aspirated under sterile circumstances, 3-5 ml was distributed in a sterile tube, and the remainder was immediately sent to the laboratory. About 2 ml bile was centrifugal by 10, 000 g for 10 min at 4°C, and then the pellet and supernatant were both kept at -80°C for the study of the biliary microbiota.

### Diagnosis of infection with *C. sinensis*

The detection of *C. sinensis* eggs in the microscopic inspection of the bile pellet could establish the infection. Firstly, 500 μL bile sample was centrifuged in eppendorf tube at 12, 000 rpm for 10 min, and then the supernatant was discarded, the bile sediment was re-suspended with 100 μL PBS. Next, 20 μL suspension was smeared and 4 smears were done for each specimen.

### DNA extraction and PCR amplification

Microbial community genomic DNA was extracted from bile sediments samples using the E.Z.N.A.DNA Kit (Omega Bio-tek, Norcross, GA, U.S.) according to manufacturer’s instructions. The DNA extract was checked on 1% agarose gel, and DNA concentration and purity were determined with NanoDrop 2000 UV-vis spectrophotometer (Thermo Scientific, Wilmington, USA). To assess the bacterial community composition, the V3-V4 region of the bacterial 16S rRNA gene was amplified by PCR (3 min of denaturation at 95°C, 27 cycles of 30 s at 95°C, 30 s for annealing at 55°C, and 45 s for elongation at 72°C, and a final extension at 72°C for 10 min), using the universal primers forward 338F (5’-ACSOCCTACGGGAGGCAGCAG-3’) and reverse 806R (5’-GGACTACHVGGGTWSOCTAAT-3’) (Fadrosh et al., 2014). The PCR mixtures contain 5 × TransStart FastPfu buffer 4 μL, 2.5 mM dNTPs 2μL, forward primer (5 μM) 0.8μL, reverse primer (5μM) 0.8μL, TransStart FastPfu DNA Polymerase 0.4μL, template DNA 10 ng, and finally ddH2O up to 20μL. PCR reactions were performed in triplicate.

### Illumina Miseq sequencing

The AxyPrep DNA Gel Extraction Kit (Axygen Biosciences, Union City, CA, USA) was used to extract the PCR product from 2% agarose gel, purify it as directed by the manufacturer, and quantify it using a QuantusTM Fluorometer (Promega, USA). Majorbio Bio-Pharm Technology Co. Ltd. used an Illumina Miseq PE300 platform (Illumina, San Diego, USA) and followed industry standard procedures to pair-end sequencing (2x300) purified amplicons (Shanghai, China) (Ling et al., 2019; Liu et al., 2019).

### Processing of sequencing data

The QIIME (v1.9.1) platform (http://qiime.org/install/index.html) was used to analyze 16S rRNA highthroughput sequencing data (http://qiime.org/install/index.html) (Caporaso et al., 2010), which could establish the 16S rRNA sequence with a similarity more than 97% as an operational taxonomic unit (OTU) and perform microbial diversity analysis (Edgar, 2010; McDonald et al., 2012). Usearch (v7.0) was used to identify and remove chimeric sequences (http://www.drive5.com/usearch/). Each 16S rRNA gene sequence’s taxonomy was analyzed by UCLUST against the Silva138 16S rRNA gene database using a 70% confidence threshold. Raw sequences were chosen based on their quality, sequence length, tag and primer. Low-quality sequences were deleted in the following manner: (1) the 300 bp readings were shortened at any location that got an average quality score of < 20 over a 50 bp sliding window, and reads shorter than 50 bp, as well as reads with unclear characters, were deleted; (2) overlapping sequences only longer than 10 bp were constructed according to their overlapped sequence. Overlap region’s maximum mismatch ratio is 0.2. Reads that could not be assembled were discarded; (3) Samples were separated based on the primers and barcode, with the sequence orientation changed, precise barcode matching and a 2 nucleotide discrepancy in primer matching.

### Bioinformatics and statistical analysis

The Quantitative Insights Into Microbial Ecology (QIIME, v 1.9.1) and R packages (v 3.2.0) were used to analyze sequencing data. The Sobs index, Shannon index, Simpson index, Ace index, and Chao1 richness estimator were used to quantify alpha diversity (within-sample diversity) for both groups, and rarefaction curves and Good’s coverage were used to assess sequence coverage (Good, 1953; Schloss et al., 2009), whereas beta diversity (between-sample diversity) was quantified by principal coordinate analysis (PCoA) plots based on weighted UniFrac distance metrics (Lozupone and Knight, 2005). The relative abundances and changes in bacterial communities for bile samples from both groups were visualized using bar graphs. To identify microbial communities at several taxonomic levels, Linear discriminant analysis effect size (LEfSe) was used to find differentially abundant taxa between groups, and the cutoff logarithmic linear discriminant analysis (LDA) score was set at 3.5 (Segata et al., 2011). The Wilcoxon rank-sum test and Metagenomeseq differential analysis were performed; the threshold for statistical significance was established at *p* < 0.05.

To evaluate the model’s diagnostic capabilities, the operating characteristic curves (receiving operational curve, ROC) were constructed and the area under the curve (AUC) was determined using IBM SPSS Statistics V25 (IBM, Armonk, NY, USA). Additionally, in order to estimate the correlations between genera biliary microbiota, and the associations between genera microbiota and clinical indicators, Spearman’s correlation coefficients were calculated using the top 30 dominant genus biliary microbiota, and Cytoscape software was used for network construction and analysis (version 3.7) (Friedman and Alm, 2012).

Furthermore, Phylogenetic Investigation of Communities by Reconstruction of Unobserved States (PICRUSt) tool was performed to predict the function of biliary microbiota (Langille et al., 2013), and the BugBase method was used for phenotype prediction of biliary microbiota (Ward et al., 2017), with statistical variance across groups examined using the Mann-Whitney-Wilcoxon test. Finally, a hierarchical clustering approach was performed using R software (version 3.5.1) to establish correlations between the biliary microbiota changes and differential metabolites.

To examine the normality of the data, the Kolmogorov-Smirnov or Shapiro-Wilk test was used. Mean and standard deviation (SD) were used to show continuous variables with normal distributions, whereas median was used to represent non-normal variables (interquartile range). A percentage was used to show categorical variables. Depending on the kind of dependent variable, binary or ordinal logistic regression was used to predict the value of biliary bacteria to clinical characteristics. Depending on the distribution of normality and the homogeneity of variance, a Student’s t-test, Welch’s t-test, or a nonparametric Wilcoxon rank-sum test was performed to determine statistical significance.

## Results

### Characteristics of patients

The demographic and clinical characteristics of all patients are shown in **Table 1**. There were no statistically significant variations with age, gender, rural life history, alanine aminotransferase (ALT), aspartate aminotransferase (AST), γ-glutamyltranspeptidase (GGT) and alkaline phosphatase (ALP) between the two groups (*P* > 0.05). While direct bilirubin (DBIL) and total bilirubin (TBIL) were significantly lower, and indirect bilirubin (IBIL) was significantly higher in *C. sinensis* infected patients (*P* < 0.05) (**Table 1**).

**Table 1:** Demographic characteristics of patients.

Characteristics	Cs*-*infected	Non-infected	*t/χ^2^/z*	*P*
Biliary obstruction	24	26		
Age, yr	55.9 ± 9.9	56.5 ± 14.3	0.167	0.868
Male	13 (54.2)	11 (42.3)	0.703	0.402
Rural life history	10 (41.7)	8 (30.8)	0.643	0.423
AST (U/L)	90.7 (35.5,282.2)	73 (42.0,115.3)	-0.583	0.560
ALT (U/L)	97.1 (25.4,273.8)	55 (39.8,139.3)	-0.874	0.382
TBIL (μmol/L)	23.0 (16.0,32.6)	66.5 (27.7,158.1)	-3.962	<0.001*
DBIL (μmol/L)	13.65 (3.8,43.6)	60 (17.4,132.6)	-2.661	0.008*
IBIL (μmol/L)	19.7 (12.0,33.2)	8.8 (5.6,15.8)	-3.515	<0.001*
ALP (U/L)	314.1 (162.9,1019.6)	247.0 (137.8,445.0)	-1.243	0.214
GGT (U/L)	504.2 (142.4,988.6)	433.0 (266,773.5)	-0.272	0.786

Results are present as medians (range, min–max)/mean ± standard deviation.

AST, aspartate aminotransferase, ALT, alanine aminotransferase, TBIL, total bilirubin, DBIL, direct bilirubin, IBIL, indirect bilirubin, ALP, alkaline phosphatase, GGT, γ-glutamyltranspeptidase.

*Statistically significant results from Mann-Whitney U test.

### Altered overall structure of biliary microbiota

**Figure 1:**
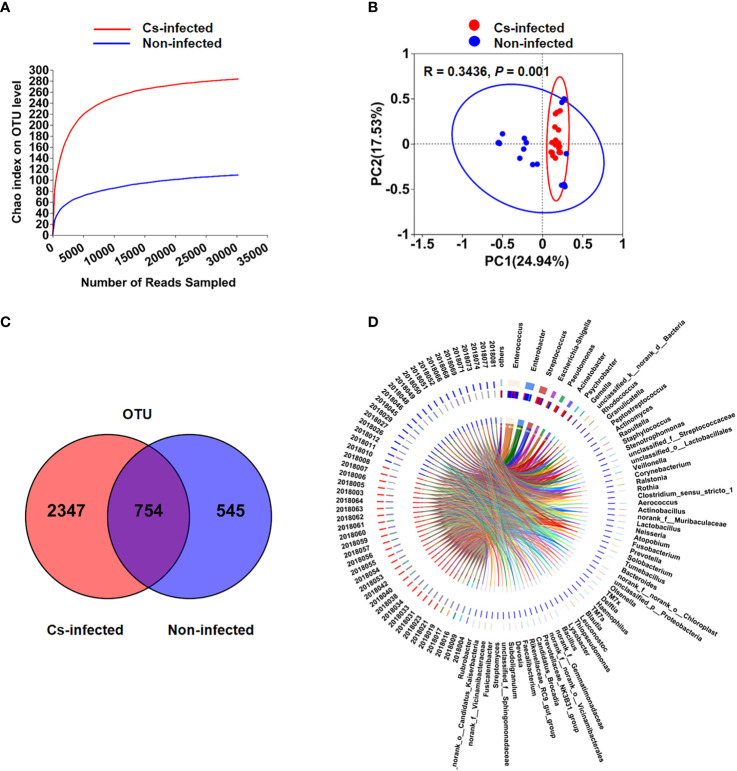
Changes in the biliary microbiota community composition and diversity of the two groups (patients infected with *C. sinensis*: n = 24; non-infected: n = 26). **(A)** Biliary microbiota rarefaction curve generated using Chao richness estimator in the two groups. **(B)** Principal coordinate analysis (PCoA) plot with Bray-Curtis distances in the two groups. The principal components PC1 and PC2 explained 24.94% and 17.53% of the variation, respectively. **(C)** The venn diagrams of OTUs between the two groups. **(D)** Circos analysis at the genus level revealed the matching abundance correlation among individual samples and microbial community structure.

Almost 2,609,499 sequences were obtained from all bile samples, after size filtering, sequence processing and quality control. These sequences were clustered into the matching OTU with 97% sequence identity. In all, 1,687 species-level phylotypes from 44 phyla, 485 families and 974 genera of biliary microorganisms were annotated for subsequent analysis. Good’s coverage score was 99.93%, suggesting that the majority of bacterial phylotypes (3,609 OTUs) in the biliary microbiota had been detected. Intriguingly, alpha-diversity indices (Shannon’s and Simpson’s indices) differed considerably between the two groups. The richness indices Sobs, ACE, and Chao1 were also significantly higher in *C. sinensis* infected group (*P* < 0.01) (**Table 2**). Additionally, the chao rarefaction curves had approached the plateau phase, indicating that species representation in individual specimens had reached a saturation point for the number of the observed species (**Figure 1A**). Meanwhile, despite considerable inter-individual differences, the bray curtis PCoA still separated the two groups into distinct clusters (ANOSIM test: R = 0.3436, *P* < 0.01; **Figure 1B**). According to the alpha and beta-diversity analyses, the overall structure of biliary microbiota had changed significantly in the *C. sinensis* infection group.

**Table 2:** Alpha diversity analysis of biliary microbiota in *C. sinensis* infected and non-infected patients.

Estimators	Cs-infected		Non-infected		*P* value
	Mean	Sd	Mean	Sd	
Sobs	2.0833	1.1765	1.3077	0.6794	0.0036
Shannon	0.5120	0.5148	0.1475	0.3138	0.0031
Simpson	0.6250	0.3505	0.8885	0.2269	0.0031
Ace	3.1840	2.9951	1.7548	1.8843	0.0228
Chao	2.7292	2.9999	1.3654	0.9334	0.0036
Coverage	0.7917	0.2603	0.9385	0.1359	0.0089

Sd, Standard Deviation.

### Composition of biliary microbiota in two groups

Differences in the composition of biliary microbiota between *C. sinensis* infected and non-infected groups were further highlighted using the Venn and Circos plots. There were 754 of the total 3,646 OTUs shared by two groups in the Venn diagram. Notably, 2,347 of the 3,101 OTU were exclusive to the *C. sinensis* infected patients, which indicating more clustering of OTUs compared to non-infected subjects (**Figure 1C**). Furthermore, Circos analysis at the genus level revealed the matching abundance association between individual samples and microbial community structure (**Figure 1D**).

**Figure 2:**
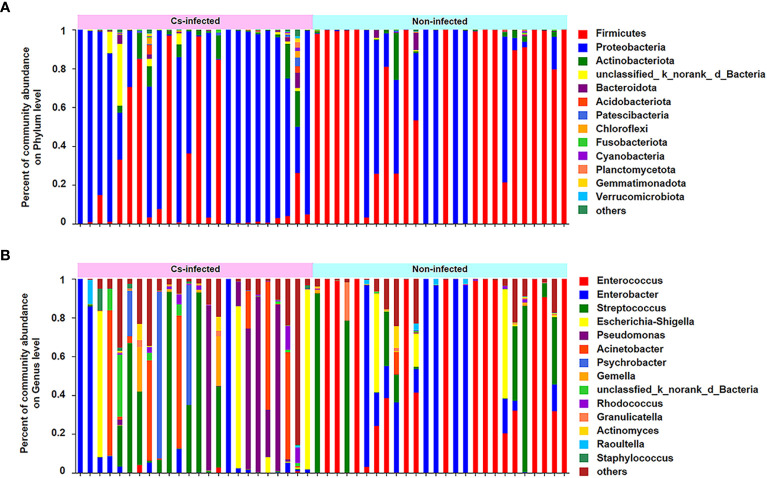
Variation of bacterial community composition of the biliary microbiota in *C. sinensis* infected (n = 24) and non-infected patients (n = 26). **(A)** The phylum level. Relative abundance < 1% are classified as others. **(B)** The genus level. Relative abundance < 10% are classified as others.

To observe the taxa composition, stacked bar plots (**Figure 2**) showed that the most abundant phylum were Firmicutes and Proteobacteria, while the *Enterococcus, Enterobacteria, Streptococcus, Escherichi, Shigella* and *Pseudomonas* dominated the biliary microbiota of the participants. The relative percentage of *Streptococcus, Escherichia, Shigella and Pseudomonas* composition were higher, while *Enterococcus* and *Enterobacteria* were lower in the *C. sinensis* infected group (**Figure 2B**). The top 30 dominant phylum and genera in all samples were selected to construct a hierarchical heatmap. Interestingly, commonly beneficial genera including *Lactobacillus* had substantially decreased, while pathogenic bacteria such as *Staphylococcus* significantly increased in the *C. sinensis* infected group (**Supplementary Figure S1**).

**Figure 3:**
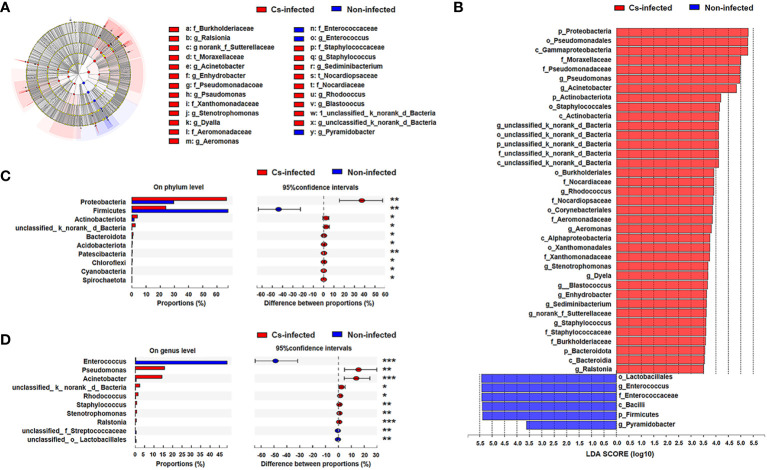
Bacterial taxa differed between *C. sinensis* infected (n = 24) and non-infected (n = 26) patients. **(A)** Taxonomic cladogram from LEfSe analysis. The size of the dots is positively correlated with the taxon’s abundance. **(B)** A histogram with linear discriminant analysis (LDA) scores based on LDA > 3.5. **(C)** Differences in bacterial taxa in the two groups at the phylum level by the Wilcoxon rank-sum test. **(D)** Difference in bacterial taxa in the two groups at the genus level by the Wilcoxon rank-sum test. Data were presented as the relative abundance (%) of phylum and genus in the two groups. Significant differences are represented by *P < 0.05, **P < 0.01, ***P < 0.001.

Next, LEfSe analysis was used to further identify the specific communities and taxonomic differences associated with *C. sinensis* infection. A cladogra illustrated the dominating bacteria and microbiota structure in the two groups (**Figure 3A**). Based on an LDA score greater than 3.5, LEfSe analysis found 37 and 6 taxa enriched in *C. sinensis* infected and non-infected patients, respectively (*P* < 0.05) (**Figure 3B**).

Additionally, we further compared the difference in specific microbial-rich taxa at phylum and genus levels by Wilcoxon rank-sum test analysis. The phyla Proteobacteria, Actinobacteriota and Bacteroidota were significantly increased, whereas Firmicutes was significantly decreased (*P* < 0.05) (**Figure 3C**); *Pseudomonas* and *Acinetobacter* genus were significantly increased, while *Enterococcus* was decreased in *C. sinensis* infected group (*P* < 0.01) (**Figure 3D**). The Metagenomeseq differential analysis found that there were 22 phylotypes differing between the two groups (*P* < 0.05). Higher abundance of most phylotypes was found in the *C. sinensis* infected subjects (i.e. absolute read counts). For example, the abundance of *Burkholderia*, *Stenotrophomonas*, and *Turicibacter* significantly elevated in *C. sinensis* infected subjects. Notablely, *Dyella*, *Aerococcus*, *Actinobacillus* and *Aggregatibacter* were only detected in *C. sinensis* infected participants (**Supplementary Table S1**).

**Figure 4:**
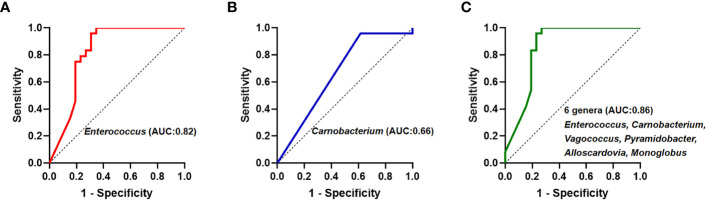
Receiver operating curve (ROC) analyses and area under the curve (AUC) of biliary microbiome data for predicting *C.sinensis* infection. **(A)** *Enterococcus* genus alone as predictor gave an AUC of 0.82 (95% CI 0.70-0.95, *P* < 0.05). **(B)** *Carnobacterium* genus alone as predictor gave an AUC of 0.66 (95% CI 0.51-0.82, *P* < 0.05). **(C)** Using the 6 genera differing between the two groups gave an AUC of 0.86 (95% CI 0.74-0.97, *P* < 0.05).

Subsequently, theses significantly different bile microbiota at the genus level were further validated to be involved in biliary obstruction and *C. sinensis* infection. ROC analyses were employed to compare the diagnostic performance of the different biliary microbiome composition data as a biomarker for *C. sinensis* infection. As shown in **Figure 4**, *Enterococcus* and *Carnobacterium* alone as predictor gave an AUC of 0.82 (95% CI 0.70-0.95, *P* < 0.05) and 0.66 (95% CI 0.51-0.82, *P* < 0.05), respectively. Combination of 6 genera including *Enterococcus, Carnobacterium, Vagococcus, Pyramidobacter, Alloscardovia, Monoglobus* have an AUC of 0.86 (95% CI 0.74-0.97, *P* < 0.05).

**Figure 5:**
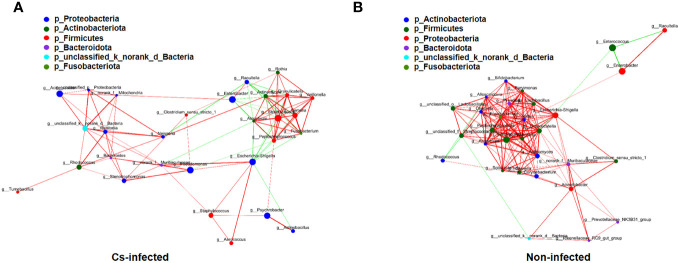
The correlations among the top 30 abundant bacterial genera were analyzed by spearman’s correlation coefficient in *C. sinensis* infected **(A)** and non-infected **(B)** groups. The node size and color indicate the relative abundance of the biliary microbiota and heritability estimates, respectively. The red line means that two nodes are positively correlated, whereas the green line means that two nodes are negatively correlated. The breadth shows the correlation’s strength (*P* < 0.05).

The structure of the bile microbiota could also be affected by dynamic interactions between these community members (Ling et al., 2020). The single factor correlation network diagram is used for the analysis of the correlation between the dominant species, which is convenient to understand the interaction between the dominant species. Therefore, correlation networks of the top 30 genera abundant microbial interaction within the groups was constructed using spearman’s analysis, respectively (**Supplementary Table S2**). Compared to *C. sinensis* infected group, non-infected group present a higher mean degree (11.33 vs. 6.90) and transitivity (0.725 vs. 0.615), indicating a more significant pairwise association between microbiota taxa of biliary aggregated (*P* < 0.05) (**Figure 5**). We found that Fusobacterium and Gemella genera have the strongest positive correlation in both two groups (r = 0.9456, *P* < 0.05), whereas Enterobacter and Enterococcus genera have the strongest negative correlation (r = -0.7444, *P* < 0.05) (**Supplementary Table S2**). A more a closer network of interactions in non-infected than that in the *C. sinensis* infected patients. These results indicate the structural dysbiosis of bile microbiota in the *C. sinensis* infected patients.

### Prediction of bacterial functional potential and microbiome phenotypes

**Figure 6:**
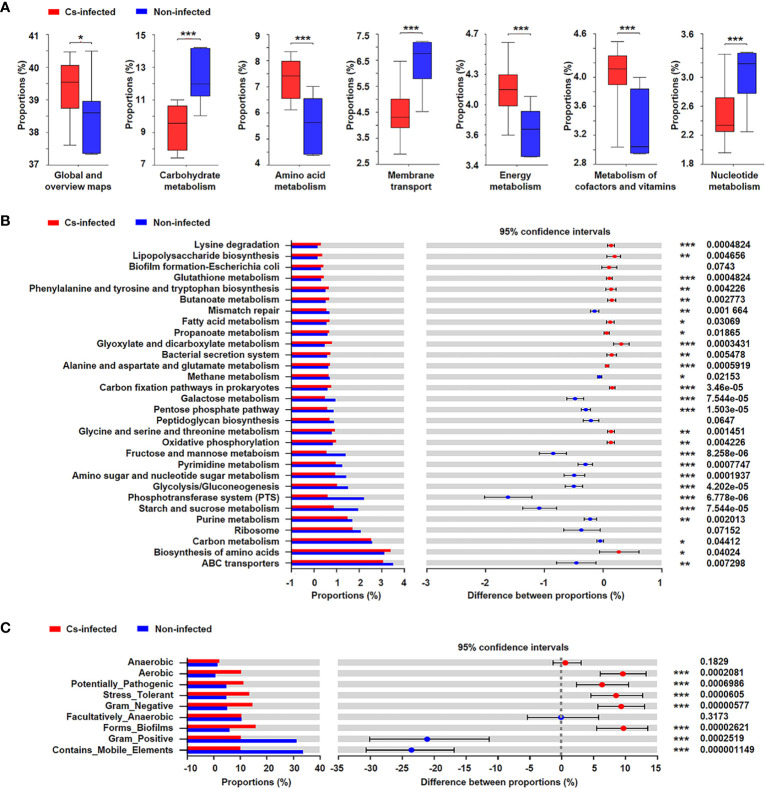
Prediction of bacterial functional potential and microbiome phenotypes in patients infected and non-infected with *C. sinensis*. **(A)** Comparative analysis of the KEGG functional category at level 2 between the two groups. **(B)** At KEGG level 3, the top 30 metabolic pathways with the highest proportion and *P* < 0.05, *Q* < 0.05 were listed based on the Wilcoxon rank-sum test. **(C)** Comparative analysis of bacterial phenotypic results based on the Wilcoxon rank-sum test. Significant differences are represented by *P < 0.05, **P < 0.01, ***P < 0.001.

The functional potential of the microbiota was predicted using PICRUSt based on 16S rRNA sequencing data. The results suggested that many KEGG level 2 pathways including amino acid metabolism, energy metabolism, carbohydrate metabolism and nucleotide metabolism (*P* < 0.05; **Figure 6A**); KEGG level 3 pathways including glyoxylate and dicarboxylate metabolism, fatty acid metabolism, oxidative phosphorylation, ABC transporters, glycolysis/gluconeogenesis (*P* < 0.05; **Figure 6B**) were significantly modulated in *C. sinensis* infected group.

In addition, the results of BugBase’s microbial phenotype prediction revealed that seven of the nine predicted phenotypic functions differed significantly between the two groups, including aerobic, potentially pathogenic, stress tolerant, forms biofilms (*P* < 0.05). Nevertheless, there was no significant difference in facultative anaerobic and anaerobic function between the two groups (*P* > 0.05) (**Figure 6C**).

### Associations between bacterial genera and differential metabolites

**Figure 7:**
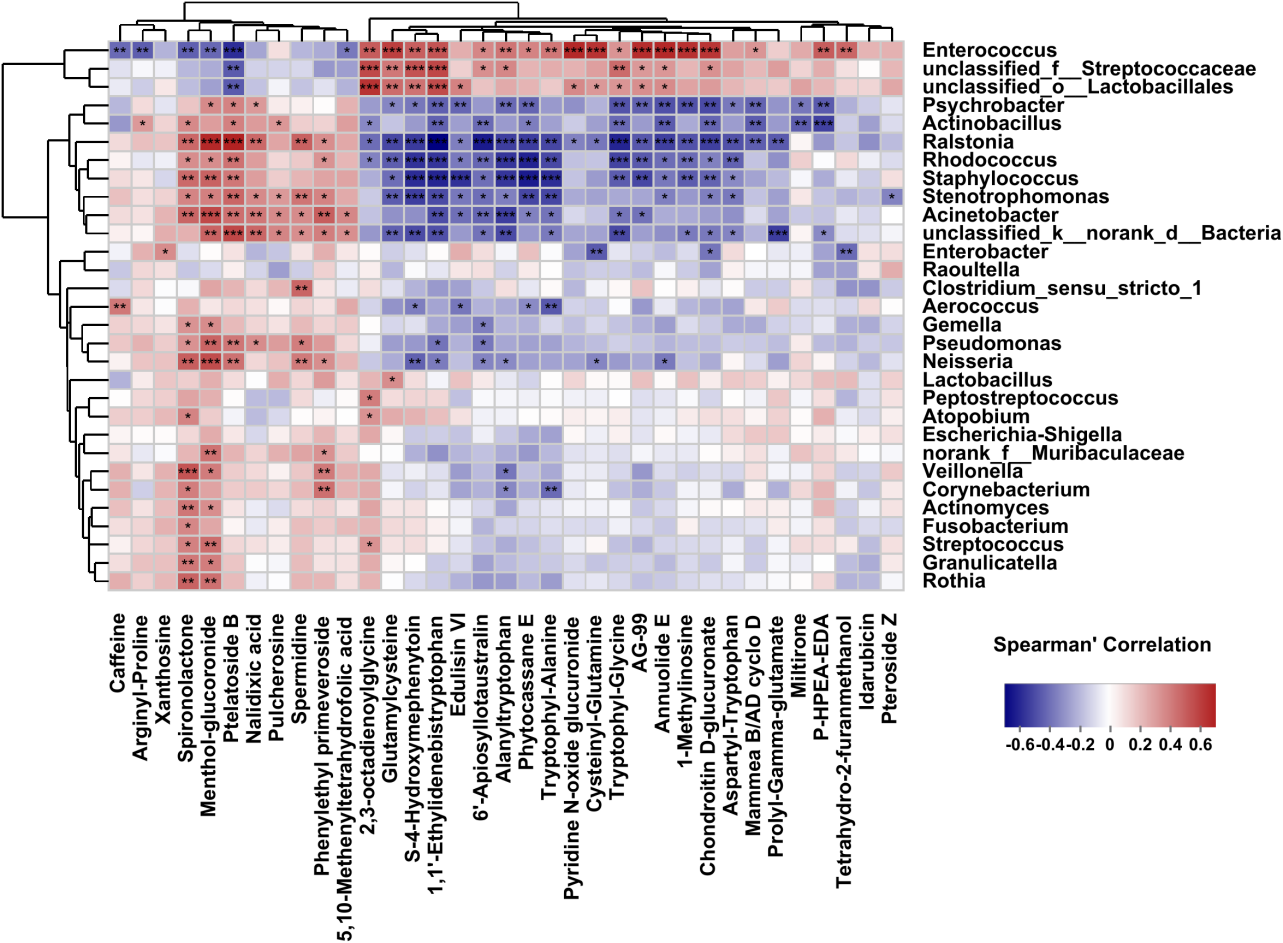
Correlations between the top 30 abundant bacterial genera and the altered 35 metabolites (VIP>2, *P*<0.05). The correlations are exhibited by colors; blue indicates a positive correlation, red indicates a negative correlation, and a darker color illustrates a stronger correlation (*p < 0.05, **p < 0.01, ***p < 0.001).

Our previous research had profiled differential metabolites based on LC-MS/MS-based metabolomics in the two groups (Zhang et al., 2023). To investigate analysis the associations between bacterial genera and differential metabolites in the host, we used Spearman’s correlation analysis to determine the covariation between the top 30 dominant biliary bacterial genera and 35 metabolites (VIP>2, *P*<0.05) in **Supplementary Table S3**, which was presented in a heatmap (**Figure 7**). Notably, the results revealed that the lower relative abundance of *Enterococcus* was positively correlated with Chondroitin D-glucuronate(r=0.63, *p*=0.00003) and Annuolide E (r=0.61, *p*=0.00001), while significantly negatively related to Ptelatoside B (r=-0.45, *p*=0.00016) in *C. sinensis* infected group (**Supplementary Table S3**). In general, these results indicate that changes in bile microbiome are related to changes in metabolites. However, we did not found some relation between the clinical indicators (TBIL, DBIL, IBIL, AST, ALT, ALP, GGT) and the microbial communities (|r| < 0.5) (**Supplementary Table S4**; **Supplementary Figure S2**).

## Discussion

Our results found that biliary microbiota in *C. sinensis* infected patients had greater α-diversity, β-diversity and more clustering of OTUs compared with the non-infected group. This finding is agree with previous research on the gut or biliary microbiota of patients infected with soil-transmitted helminths (Lee et al., 2014; Saltykova et al., 2016; Bao et al., 2018; Xu et al., 2018). However, the influence of helminth infection on the biliary microbial diversity remains controversial (Saltykova et al., 2018). In other studies, increasing of alpha diversity was not evident in patients infected with parasites (Cooper et al., 2013; Kim et al., 2019; Hu et al., 2020). These differences might be attributed to variances with geographical location, platform used, parasites infection state and sequencing depth (Xu et al., 2018). In addition, our findings also revealed considerable inter-individual variance in the biliary microbiota, which is consistent with previous research (Chen et al., 2019). It might been related to host lifestyles, age, disease status and dietary pattern, which influence the bacterial colonization or survival (Ye et al., 2016; Chen et al., 2019). In order to avoid the confounding factors influenced group discrimination, the variables of the two groups were generally matched in the study, and there were no statistically significant variations with the average age, similar lifestyle or dietary pattern in two groups. However, age, lifestyle or dietary pattern also influence the composition of biliary microbiome within groups, which induce the inter-individual variance.

Similar to previous studies on biliary microbiota (Saltykova et al., 2016; Ye et al., 2016; Liu et al., 2020; Han et al., 2021), our study also found the dominant phylum in bile samples including Proteobacteria, following with Firmicutes, Actinobacteria, Bacteroidetes, etc. Based on the bile microbiota from healthy individual, it seems that theses 3 to 4 phyla constitute the core population of healthy human bile using 16S rRNA gene profile analysis (Molinero et al., 2019). It is suggested that the impact of infection on microbial structure at phyla does not seem to be as significant on the background of biliary obstruction. However, Proteobacteria was found highly enriched *C. sinensis* infected patients. Proteobacteria, as a Gram-negative phylum, includes many important pathogens like Salmonella, Vibrio and Helicobacter, which can produce a variety of neurotoxins (Mladenova-Hristova et al., 2017; Yan et al., 2021). The abnormal increase of Proteobacteria is related to increased epithelial oxygen availability and considered as a biomarker of inflammation and epithelial dysfunction (Litvak et al., 2017). However, less is known about how the increased Proteobacteria links with the inflammation and morbidity after parasite infection. The role of Proteobacteria in biliary microbiota of *C. sinensis* infected patients needs further study.

At the genus level, the most dominant genus was *Enterococcus*, followed by *Pseudomonas, Acinetobacter, Rhodococcus, Staphylococcus*, etc., most of which belong to Proteobacteria and Firmicutes. Among them, the abundance of *Pseudomonas* and *Staphylococcus* genera were significantly increased in the *C. sinensis* infected group, which was observed in several hepatobiliary diseases (Grigor'eva and Romanova, 2020; Liu et al., 2020; Ye et al., 2020). Some subspecies of *Staphylococcus* have highly virulent pathogens and multiple antibiotic-resistance (Plata et al., 2009; Gardete and Tomasz, 2014). The role of increased pathogen genera of microbiota associated with clonorchiasis and associated hepatobiliary tract disorders can be expected to be informative with respect to fibrosis and carcinogenesis. Thus, we believe that the potential functional and prognostic role of *Pseudomonas* and *Staphylococcus* in the *C. sinensis* infected group should be studied in the intestine and the bile ducts in future studies. *Enterococcus* is part of the natural flora of the human gut, and also survive in bile-rich environments (Liu et al., 2020). It has maintaining gut flora balance, immune regulatory and anti-allergic properties function (Bodera and Chcialowski, 2009). The lower relative abundance of *Enterococcus* in the bile of *C. sinensis*-infected group might lead to a disruption of the inter-dependent balance of bile flora. Theses relevant differences at genera level between the two groups are depending on the specific physiological condition of the host, such as infection.

Further functional analysis showed that amino acid metabolism pathways was significantly enriched and changed in *C. sinensis* infected patients, as reported in other research on the *Opisthorchis viverrini* associated microbiota dysbiosis (Chng et al., 2016). We noticed that the function of the differential flora was similar with the metabolic pathway enriched by the differential metabolites. Some of the pathways were supported by subsequent LC/MS-based metabolomics analyses. The enrichment of Amino acid metabolism may lead to ammonia production increased as a side product (Saltykova et al., 2018). Increased of these potentially carcinogenic metabolite may promote the malignant transformation of clonorchiasis (Chng et al., 2016). Furthermore, our results revealed the decreased expression of ABC transporters and Lipopolysaccharide biosynthesis enhanced in *C. sinensis*-infected patients. The findings are consistent with research on these pathways implicated in inflammation and hepatobiliary disorders (Han et al., 2021; Hao et al., 2022). Bacterial ABC transporters can protect microorganisms from exogenous stress (Fath and Kolter, 1993). The apparent downregulation of ABC transporters might implicate a decreased antimicrobial pressure in the biliary environment (Wang et al., 2019). On the other hand, the enhanced of Lipopolysaccharide biosynthesis may drive biliary epithelial inflammation in *C. sinensis*-infected patients (Karrar et al., 2007). These results point a contribution of altered biliary microbiota to cholangiocyte and bile duct mucosal damage (Liwinski et al., 2020). Although we have done a combined analysis of microbiota and metabolism, we could not confidently assign individual metabolites to individual microbial species. Furthermore, other additional metabolite sources could not be rule out, which may also cause differ between patients infected with C. *sinensis* and non-infected patients. The metabolite changes might be microbiota dependent but not necessarily microbiota derived (Cheema and Pluznick, 2019). Taken together, alteration of biliary microbiota caused by *C. sinensis* infection may be involved in the alteration of host metabolism pathway. We speculate that the interaction of *C. sinensis*, Biliary Microbiota and related metabolites may be an important target for research on biliary obstruction.

This study provides new perspectives on the microbiological characteristic of biliary obstruction patients with *C. sinensis* infection. However, it also has several limitations. Firstly, due to ethical and technological challenges, it is difficulty to collected the bile from healthy subjects. Because of different amplified and sequenced area, some healthy individual biliary microbiota data cannot be compared with our s. Although some sequence data of bile from healthy individuals are provided in some research, it is more reasonable to obtain samples from Chinese patients, taking into account the factors such as diet and race (Han et al., 2021). Secondly, microbiome analysis was restricted to a single-center cohort of patients who had the same race and dietary habits in northern China. In the future, multicenter study cohorts should be considered to determine the stability of observed alterations in biliary microbiota. Thirdly, to reduce antibiotic interference with biliary microbiota, patients who did not receive antibiotics 6 months prior were selected. However, the antibiotic impact cannot be ruled out altogether. Further validation of differential pathways at the functional level using metagenomes and follow-up experiments for suggested pathways and the expression levels of crucial difference genes will be good evidence of what is happening in the biliary tract. Fourthly, because of many underlying causes of biliary obstruction, the biliary obstruction without infection patients might represent variations in their microbial content based on these underlying factors. These factors need to be considered. Finally, because it is difficult to evaluate the worm burden of the patients, the correlations between the worm burden and the dysbiosis of biliary microbiota were not evaluated. Even considering these limitations, our results establish the basis for future larger-scale studies on the relationship between bile microbiota, parasite infection and diseases.

## Conclusion

In summary, biliary obstruction patients infected with *C. sinensis* modified the biliary microbiome, compared to non-infected subjects. The abundant beneficial bacteria genera such as *Enterococcus* decreased prominently, while abundant conditional pathogen genera, such as *Pseudomonas, Acinetobacter, Rhodococcus, Staphylococcus*, and *Stenotrophomonas* increased significantly in *C. sinensis* infected patients. These results could supply novel information for further understanding of the pathogenic mechanism of biliary injury following *C. sinensis* infection. Additionally, the pathogenic mechanism involved in biliary microbiota and clonorchiasis need to be further explored.

## Data availability statement

The datasets presented in this study can be found in online repositories. The names of the repository/repositories and accession number(s) can be found in the article/**Supplementary Material**.

## Ethics statement

The studies involving humans were approved by Committee of Harbin Medical University (Harbin, China). The studies were conducted in accordance with the local legislation and institutional requirements. The participants provided their written informed consent to participate in this study.

## Author contributions

RC: Conceptualization, Data curation, Validation, Visualization, Writing – original draft, Writing – review & editing, Formal analysis, Methodology. XLi: Conceptualization, Data curation, Validation, Visualization, Writing – original draft, Writing – review & editing, Formal analysis, Investigation, Methodology, Software. JD: Data curation, Investigation, Methodology. JW: Data curation, Investigation, Methodology. XuZ: Data curation, Investigation, Methodology. XJ: Data curation, Investigation, Methodology. SD: Data curation, Investigation, Methodology. XH: Data curation, Investigation, Methodology. YG: Data curation, Investigation, Methodology. BS: Data curation, Investigation, Methodology. XLu: Methodology, Resources. RW: Methodology, Resources. YC: Writing – review & editing, Validation. XiZ: Supervision, Validation, Writing – review & editing, Conceptualization, Data curation, Formal analysis, Funding acquisition, Methodology, Project administration, Visualization, Writing – original draft. SH: Conceptualization, Data curation, Formal analysis, Funding acquisition, Project administration, Supervision, Validation, Visualization, Writing – review & editing, Investigation, Resources.

## Supplementary material

Supplementary Figure 1 Hierarchical clustering of biliary microbiota in *C. sinensis* infected (n = 24) and non-infected patients (n = 26). **(A)** The phylum level. **(B)** The genus level. Red, high abundance; blue, low abundance.

Supplementary Figure 2 Correlation analysis between relative abundance of biliary microbiota and clinical parameters in *C. sinensis* infected (n = 24) and non-infected patients (n = 26) were performed by using Spearman’s correlation analysis. The color of the spots in the right panel represents R-value of Spearman’s correlation between the genera and clinical parameters; positive correlations are represented by red, negative correlations are represented by blue, and a darker color represents a stronger correlation. Significant differences are represented by **P* < 0.05, ***P* < 0.01, ****P* < 0.001. AST, aspartate aminotransferase, ALT, alanine aminotransferase, TBIL, total bilirubin, DBIL, direct bilirubin, IBIL, indirect bilirubin, ALP, alkaline phosphatase, GGT, γ-glutamyltranspeptidase.

Supplementary Table 1 Numerous phylotypes of the biliary microbiota differed between in Cs-infected and Non-infected groups.

Supplementary Table 2 The correlation coefficient matrix parameters among the top 30 abundant bacterial genera by Spearman’s analysis in Cs-infected and Non-infected groups, respectively.

Supplementary Table 3 The correlation coefficient matrix between the top 30 abundant bacterial genera and the significant altered 35 metabolites (VIP>2, P<0.05) by Spearman's analysis in Cs-infected and Non-infected groups.

Supplementary Table 4 The correlation coefficient matrix between bacterial genera and significantly different clinical parameters using Spearman’analysis in Cs-infected and Non-infected groups.
