# Supplementary figures and images for: Profiles of biliary microbiota in biliary obstruction patients with Clonorchis sinensis infection
Source: Front Cell Infect Microbiol. 2023 Dec 18;13:1281745. doi: 10.3389/fcimb.2023.1281745 (PMC10757933; doi:10.3389/fcimb.2023.1281745)

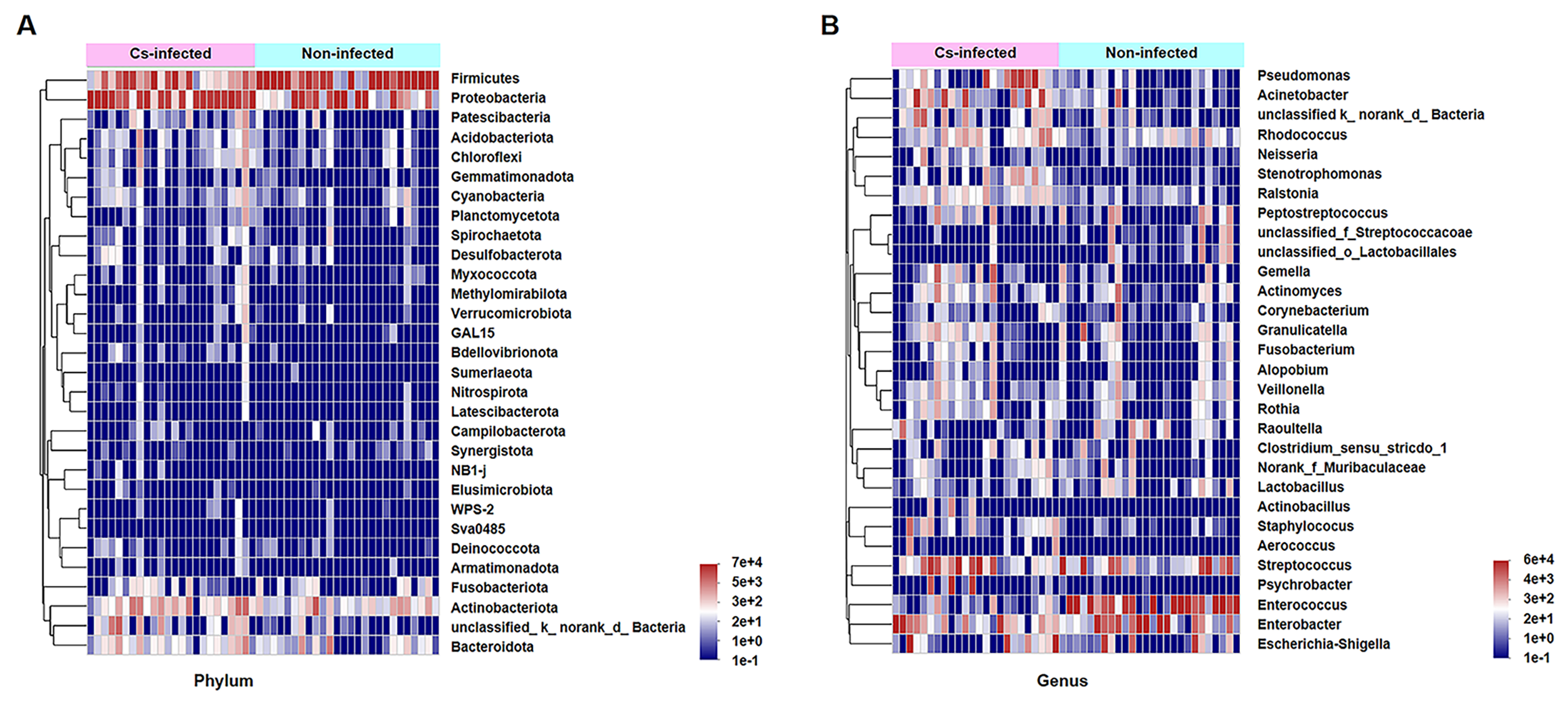

Supplement: Supplementary Figure 1 — Hierarchical clustering of biliary microbiota in C. sinensis infected (n = 24) and non-infected patients (n = 26). (A) The phylum level. (B) The genus level. Red, high abundance; blue, low abundance. [file Image_1.tif]

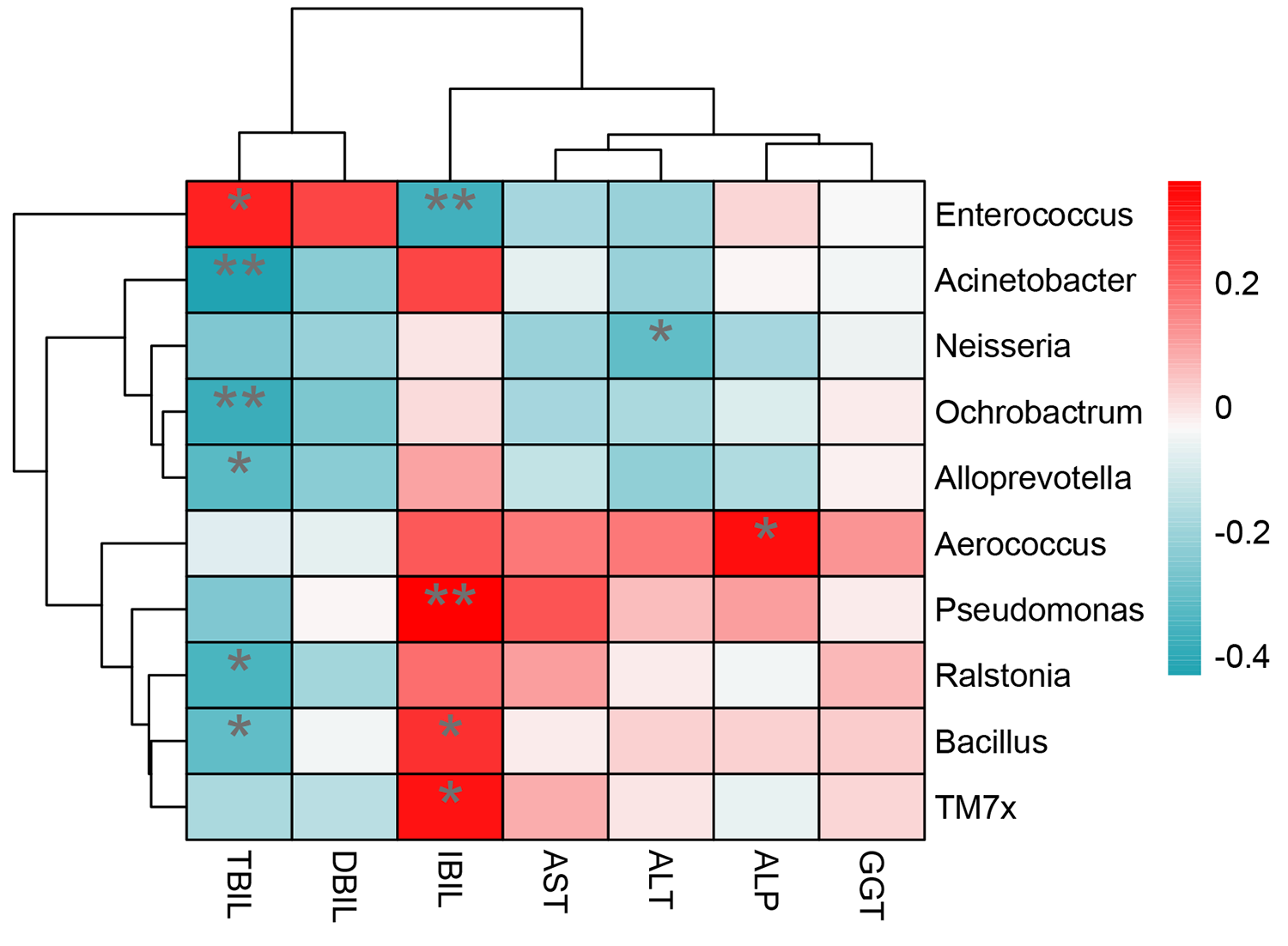

Supplement: Supplementary Figure 2 — Correlation analysis between relative abundance of biliary microbiota and clinical parameters in C. sinensis infected (n = 24) and non-infected patients (n = 26) were performed by using Spearman’s correlation analysis. The color of the spots in the right panel represents R-value of Spearman’s correlation between the genera and clinical parameters; positive correlations are represented by red, negative correlations are represented by blue, and a darker color represents a stronger correlation. Significant differences are represented by *P < 0.05, **P < 0.01, ***P < 0.001. AST, aspartate aminotransferase, ALT, alanine aminotransferase, TBIL, total bilirubin, DBIL, direct bilirubin, IBIL, indirect bilirubin, ALP, alkaline phosphatase, GGT, γ-glutamyltranspeptidase. [file Image_2.tif]
