# Supplementary material for: Profiles of biliary microbiota in biliary obstruction patients with Clonorchis sinensis infection
Source: Front Cell Infect Microbiol. 2023 Dec 18;13:1281745. doi: 10.3389/fcimb.2023.1281745 (PMC10757933; doi:10.3389/fcimb.2023.1281745)
Supplement: Supplementary Table 1 — Numerous phylotypes of the biliary microbiota differed between in Cs-infected and Non-infected groups. [file Table_1.docx]

**Table S1.** Numerous phylotypes of the biliary microbiota differed between in Cs-infected and Non-infected groups.

| Genus | Mean of reads | | *p* value | % of samples that contain reads | |
| --- | --- | --- | --- | --- | --- |
|  | Cs-infected | Non-infected |  | Cs-infected | Non-infected |
| *Burkholderia* | 30.67 | 2.96 | 0.0001 | 79.17 | 26.92 |
| *Stenotrophomonas* | 319.33 | 1.65 | 0.0002 | 62.50 | 26.92 |
| *Turicibacter* | 3.96 | 0.12 | 0.0002 | 37.50 | 3.85 |
| *Bacillus* | 44.38 | 0.15 | 0.0007 | 58.33 | 7.69 |
| *Caulobacter* | 4.38 | 0.04 | 0.0007 | 50.00 | 3.85 |
| *Pseudomonas* | 6163.79 | 15.42 | 0.0007 | 79.17 | 53.85 |
| *Carnobacterium* | 4.33 | 3.88 | 0.0009 | 4.17 | 38.46 |
| *Staphylococcus* | 485.71 | 4.08 | 0.0010 | 70.83 | 38.46 |
| *Vagococcus* | 3.04 | 1.77 | 0.0016 | 4.17 | 38.46 |
| *Dyella* | 0.96 | 0.00 | 0.0023 | 33.33 | 0.00 |
| *Pyramidobacter* | 0.13 | 1.27 | 0.0024 | 4.17 | 26.92 |
| *Aerococcus* | 163.54 | 0.00 | 0.0026 | 20.83 | 0.00 |
| *Acinetobacter* | 5503.33 | 260.88 | 0.0033 | 87.50 | 26.92 |
| *Actinobacillus* | 159.58 | 0.00 | 0.0037 | 20.83 | 0.00 |
| *Pelomonas* | 2.46 | 0.35 | 0.0041 | 45.83 | 53.85 |
| *Aggregatibacter* | 1.71 | 0.00 | 0.0058 | 29.17 | 0.00 |
| *Psychrobacter* | 3575.75 | 0.23 | 0.0063 | 29.17 | 11.54 |
| *Alloscardovia* | 0.00 | 26.58 | 0.0065 | 0.00 | 15.38 |
| *Enhydrobacter* | 2.00 | 0.12 | 0.0074 | 29.17 | 7.69 |
| *Monoglobus* | 0.00 | 0.96 | 0.0075 | 0.00 | 11.54 |
| *Rhodobacter* | 8.17 | 0.04 | 0.0086 | 33.33 | 3.85 |
| *Anoxybacillus* | 0.04 | 3.42 | 0.0087 | 4.17 | 15.38 |
